# Stem loop-mediated isothermal amplification test: comparative analysis with classical LAMP and PCR in detection of *Entamoeba histolytica* in Kenya

**DOI:** 10.1186/s13104-017-2466-3

**Published:** 2017-03-31

**Authors:** Fridah Mwendwa, Cecilia K. Mbae, Johnson Kinyua, Erastus Mulinge, Gitonga Nkanata Mburugu, Zablon K. Njiru

**Affiliations:** 1 Institute of Tropical Medicine and Infectious Diseases, Jomo Kenyatta University of Agriculture and Technology, P.O Box 62000-0200, Nairobi, Kenya; 2 Centre for Microbiology Research, Kenya Medical Research Institute, P.O Box 19464-00202, Nairobi, Kenya; 3 Department of Biochemistry, Jomo Kenyatta University of Agriculture and Technology, P.O Box 62000-0200, Nairobi, Kenya; 4 Meru University of Science and Technology, P. O. Box 972-60200, Meru, Kenya; 5 School of Health Professions, Mandurah Campus, Murdoch University, Murdoch, WA 6210 Australia

**Keywords:** Amoebiasis, *Entamoeba histolytica*, Diagnosis, Loop-mediated isothermal amplification, LAMP, Stem-LAMP test, Kenya

## Abstract

**Background:**

*Entamoeba histolytica*, the causative agent for amoebiasis is a considerable burden to population in the developing countries where it accounts for over 50 million infections. The tools for detection of amoebiasis are inadequate and diagnosis relies on microscopy which means a significant percent of cases remain undiagnosed. Moreover, tests formats that can be rapidly applied in rural endemic areas are not available.

**Methods:**

In this study, a loop-mediated isothermal test (LAMP) based on 18S small subunit ribosomal RNA gene was designed with extra reaction accelerating primers (stem primers) and compared with the published LAMP and PCR tests in detection of *E. histolytica* DNA in clinical samples.

**Results:**

The stem LAMP test indicated shorter time to results by an average 11 min and analytical sensitivity of 10^−7^ (~30 pg/ml) compared to the standard LAMP and PCR which showed sensitivities levels of 10^−5^ (~3 ng/ml) and 10^−4^ (~30 ng/ml) respectively using tenfold serial dilution of DNA. In the analysis of clinical specimens positive for *Entamoeba* spp. trophozoites and cysts using microscopy, the stem LAMP test detected *E. histolytica* DNA in 36/126, standard LAMP test 20/126 and PCR 17/126 cases respectively. There was 100% agreement in detection of the stem LAMP test product using fluorescence of SYTO-9 dye in real time machine, through addition of 1/10 dilution of SYBR^®^ Green I and electrophoresis in 2% agarose gel stained with ethidium bromide.

**Conclusion:**

The stem LAMP test developed in this study indicates potential towards detection of *E. histolytica*.

## Background

Amoebiasis caused by protozoan *Entamoeba histolytica* is an important human gastrointestinal infection responsible for over 50 million amoebic infection cases with over 100,000 deaths annually [1]. It is a leading cause of death only surpassed by malaria and schistosomiasis [2] with most of these cases being reported in the developing countries [3–5]. In Africa, the burden of amoebiasis is high with an estimated *E. histolytica* infection median rate of 796 per 100,000 people [6]. Studies conducted in Kenya indicated prevalence of 6–11% of *E. histolytica*/*Entamoeba dispar* in children at selected hospitals [4, 5, 7] and 11–32% among adults [8]. Moreover, in more recent studies, the prevalence of *E. histolytica* by qPCR was recorded at 15% in Bungoma County, Western Kenya [9] while a much lower prevalence of 0.4% was reported among children with vertically transmitted HIV infection [10]. This data suggests that amoebiasis is a heavy burden among Kenyan population which is also plagued by other diseases such as malaria, HIV-AIDS, tuberculosis and other non-communicable diseases.

The *E. histolytica* infection causes several intestinal and extra-intestinal conditions with dysentery and liver abscess being the most common [11]. The algorithm to diagnose amoebiasis is often complex due to the unsatisfactory sensitivity and specificity of available tests. Microscopy is widely used but has low sensitivity and cannot differentiate *E. histolytica* from the morphologically similar non-pathogenic species *E. dispar* and amphizoic *E. moshkovskii* [12]. Stool culture followed by isoenzyme analysis has been used to differentiate species but the methods are time consuming hence impractical for use in the routine diagnosis. Antibody detection tests have been developed and used widely but their downturn is low sensitivity in early disease and inability to distinguish active infection from previous exposure [13]. The *E. histolytica* antigen detection in the stool using ELISA tests [14–16] has proved more sensitive than microscopy, however cross-reactivity with *E. dispar* limit their application [17, 18]. This far the PCR method has been the most sensitive method for discriminating between *E. histolytica* and *E. dispar*. Indeed, several PCR tests have been developed [19–21] but despite the reported advantage of PCR tests in diagnosis of *E. histolytica,* the method has limited use in routine diagnosis of amoebiasis in Kenya due to associated cost.

In the last decade, a rapid DNA amplification test called loop-mediated isothermal amplification (LAMP) of DNA was developed [22]. The technique is a novel strategy for gene amplification which relies on DNA polymerase with strand displacement activities. The LAMP technique has recently been applied in detection of human diseases such as malaria [23, 24], human toxoplasmosis [25] and meningitis [26] and has been hypothesized to revolutionize field based molecular test [27, 28]. The LAMP is well suited for *E. histolytica* diagnosis in endemic areas because it does not require expensive equipment to achieve amplifications, sensitivity is equivalent to that of PCR and time to results is approximately 1 h. Moreover, the large amount of products formed offers the use of different visual detection formats that are applicable in rural endemic areas. Indeed, LAMP has recently been used successfully to detect other human stool pathogens such as *Ascaris lumbricoides* [29], *Clostridium difficile* [30] and hookworms [31]. Previously, LAMP tests for *E. histolytica* have been developed based on small subunit rRNA gene [32] and HLY6 gene [33]. In 18S rRNA gene, the nested PCR detected 0.1–1 parasite per reaction compared to LAMP test which detected 1 parasite [32] and 2 ng/µl compared to 15.8 ng/µl (~5 parasites per reaction) for LAMP test using DNA for HLY6 target respectively [33]. LAMP uses many reaction components which is a major cost in the developing countries [34]. However, several companies have come up with ready to use commercial isothermal master mixes slightly reducing the cost burden and need for protracted optimization procedures. These include Optigene, UK (http://www.optigene.co.uk/products-reagents/) and EIKEN Chemical Co Ltd, Japan (http://www.eiken.co.jp/en/). The advantages presented by LAMP method as a potential point of use test calls for more attention in improving this platform for use in endemic countries. On this context, [35] reported improved amplification speed and sensitivity of *C. difficile, Listeria monocytogenes* and HIV LAMP tests through addition of a second reaction accelerating primers called stem primers (target the stem section of the LAMP amplicon). In addition, stem primers have been used recently to improve the sensitivity of *Trypanosoma brucei gambiense* LAMP test by ~100-fold compared to the standard LAMP test [36]. The advantage of stem primers is that they can be used in multiplex with loop primers [37] without affecting test reproducibility. In this work, we report an improved LAMP test for *E. histolytica* with inclusion of stem primers.

## Methods

### Reference DNA

The reference DNA sample of *E. histolytica* HM-1: IMSS was kindly provided by Dr. Graham Clark, Department of Pathogen Molecular Biology, London School of Hygiene and Tropical Medicine, UK. The DNA to check the test specificity was prepared from *E. dispar* and *Giardia lamblia* using commercial DNA extraction kit (Qiagen, Essex, UK).

### Clinical samples

All samples were collected from children who presented to three participating outpatient clinics and those admitted to the paediatric ward of Mbagathi District hospital, Nairobi were examined for the presence of *E. histolytica*. In order to improve sensitivity of microscopy in detection of *E. histolytica* cysts, the technique of formal-ether concentration was applied [38].

### DNA extraction

The DNA was prepared from 126 samples scored as positive for Entamoeba (*E. histolytica*, *E. dispar* and *E. moshkovskii* complex) using microscopy. Genomic DNA was extracted using QiAmp^®^ DNA stool Mini kit (Qiagen, Crawley, West Sussex, United Kingdom) as per the manufacturer’s instructions with slight modifications. Briefly, 200 μl of fecal suspension was washed five times with distilled water. To this suspension, 1.4 ml of ASL buffer was added and subjected to five times thawing (80 °C) and freezing (−80 °C) to rupture the rigid cysts. The genomic DNA was eluted in 50 μl of nuclease-free water and stored at −20 °C until use.

### PCR test

The PCR test targeting the small-subunit rRNA gene was used [20] with some modifications. Briefly a 25 µl test was done and consisted of 1× PCR buffer, 1.5 mM MgCl_2_, 2 mM dNTPs, 0.5 U of *Taq* polymerase and 10 pmol of forward primer (EntaF) and reverse primer (EhR). These primers generate a 166-bp PCR product and are specific for *E. histolytica*. The reference DNA template was 2 µl of DNA and 3–4 μl for clinical samples. The amplifications were done in a PCR system 9700 thermal cycler (Applied Biosystems, UK) under the following cycling conditions: An initial denaturation step at 94 °C for 3 min, followed by 35 cycles each consisting denaturation at 94 °C for 1 min, annealing at 58 °C for 1 min and extension at 72 °C for 1 min. The final extension was at 72 °C for 7 min. Reactions were done in duplicates and the resulting amplification products were separated by electrophoresis in 2.0% agarose gel in 1 × Tris–borate-EDTA at 100 V for 45 min and visualized under UV light after staining with ethidium bromide.

### Design of LAMP primers

Four sets of primers each recognizing ten distinct sections of *E. histolytica* 18S small subunit ribosomal RNA (18S rRNA gene) (Genbank accession number X64142) and hemolysin (HLY6) gene (GenBank accession number Z29969.1) were designed using Primer Explorer version 3 software (http://primerexplorer.jp/lamp3.0.0/index.html). The targets were chosen due to the reported specificity and high number of copies (~200 copies) for18S rRNA gene [39] and HLY6 (400 copies/cell) [33]. The software designed the following primers: forward and backward outer primers (F3 and B3) and forward and backward inner primers (FIP and BIP). The loop forward and backward primers (LF and LB) and stem forward and backwards primers (SF and SB) were manually designed following the respective published primer characteristics [22, 35]. The primers were blasted for target specificity using the basic local alignment search tool (http://www.ncbi.nlm.nih.gov/BLAST). The designed tests consisted of F3/B3, FIP/BIP, LF/LB and SF/SB primer combination.

### LAMP reactions

The 18S and HLY6 LAMP primers were first analyzed for detection of the reference *E. histolytica* HM-1: IMSS using standard LAMP conditions. The tests specificity was checked with closely associated pathogen DNA extracted from morphologically similar but non-pathogenic *E. dispar* and *G. lamblia.* The primer set(s) that passed these criteria were then analyzed using a tenfold serial dilution of control DNA and using the standard LAMP test conditions [22]. The most sensitive primer set for each target was selected for further analysis. The new tests were labeled stem 18S and Stem HLY6 LAMP tests respectively (Table 1) and the selected primer sets were used to optimize respective LAMP test using Taguchi method [40]. Briefly, four reaction components determined to have the greatest effect on LAMP reaction namely inner primers, loop primers, stem primers and dNTPs had their concentrations varied at three levels. The inner primer concentration was varied from 30 to 60 pmol, loop primers from 10 to 30 pmol, stem primers from 10 to 40 pmol and dNTPs from 1 to 3 mM respectively. The concentrations of each reaction component were arranged in an orthogonal array [40] and used to determine the amount of amplification product formed [40]. This was followed by regression analysis to determine the concentration optima for each selected reaction component [40]. Other reaction components included 1× ThermoPol reaction buffer contained 20 mM Tris–HCl (pH8.8), 10 mM KCl, 10 mM (NH_4_)_2_SO_4_, 2 mM MgSO_4_ and 0.1% Triton X-100. The *Bst* 3.0 DNA polymerase (New England Biolabs, MA USA) was 0.5 µl, betaine at 0.8 M and SYTO-9 fluorescence dye at 2.0 µM (Molecular Probes, Oregon, USA). The template was 2 µl of DNA. The LAMP reaction were performed for 60 min at 62 °C using the real-time PCR machine and data acquired on FAM channel followed by reaction inactivation at 80 °C for 5 min. Once the optimized reaction conditions were determined the reactions were duplicated using a thermocycler and a water bath that maintained temperature at ~61–63 °C. The template for clinical samples was varied from 2 to 4 µl. For comparative purposes, the published LAMP test based on small subunit rRNA gene [32] and HLY6 LAMP test [33] were included.

**Table 1:** Nucleotide sequences for *E. histolytica* primers for stem LAMP test based on 18S rRNA and HLY6 genes

Target	Primer name	Sequence (5′–3′)	Bases	Final amplicon size
18S rRNA gene	F3	AAATACAAGGATAGCTTTGTG	21	
	B3	AAGCTCCCTCTCCGATGTC	19	
	FIP	CTCAATTCATTGAATGAATTGGCATGATAAAGATAATACTTGAGAC	46	207
	BIP	CAATGAGAATTTCTGATCTATCCGTTATCCGTTATAATCTTGG	43	
	LF	TTTGTACTAATACAAACTGGATC	23	
	LB	CAGTTGGTAGTATCGAGGAC	20	
	SF	CGACAATTGTAGAACACACAG	21	
	SB	ATCCTAACTCACTTAGAATGTC	22	
HLY6	F3	ATACTTGAACGGATTG AAGCC	21	
	B3	GTTTATTCATATGTTTGACAAGA	22	
	FIP	CGCCCTATACTCAAATATGACACTTTGGTGGAAGATTCACG	41	190
	BIP	AGGAAGGTCAAAGTATTAATAGTGTTGAGTGAATATACTCACC	43	
	LF	GTAATTTGCACGTTAACACTG	21	
	LB	TGGTAAAGATAATGATTAGGTG	22	
	SF	CTGGTTCCACCTGAATATTC	20	
	SB	TACTAGATAGTTCGATGAGTC	21	

### Detection and confirmation of LAMP product

The LAMP product was detected through fluorescence of SYTO-9 dye in real time PCR machine, through electrophoresis in 2% agarose gel stained with ethidium bromide and after addition of 1 µl of 1/10 dilution of 10,000× stock SYBR^®^ Green I (Sigma-Aldrich, St. Louis, MO, USA). To confirm that *E. histolytica* LAMP test amplified the predicted product, melt peaks were acquired using 1 °C steps, with a hold of 30 s, from 62 to 96 °C [41] post amplification and through digestion of the resulting LAMP product using restriction enzyme *Dde*I (New England BioLabs, MA, USA) and following manufacturers recommendations.

### Analytical sensitivity LAMP test

The analytical sensitivity for the stem 18S LAMP test was carried out in duplicates using a tenfold serial dilution of ~300 µg/ml of reference DNA from *E. histolytica* DNA. To cover different published LAMP tests formats [32, 33, 35]. The following primer combinations were used: (i) Stem LAMP test with outer primers, (ii) Stem LAMP test without outer primers, (iii) Standard LAMP with loop primers and (iv) Standard LAMP test without loop primers (Table 2). These formats were compared with published LAMP test (without loop primers) [32] and PCR targeting 18S rRNA gene [20].

**Table 2:** The analytical sensitivity of LAMP tests based on 18S rRNA gene and PCR using a tenfold serial dilution of *E. histolytica* DNA

Test	Combination	Tenfold serial dilution						C_*T*_ value	Remarks
		Neat	10^−1 to 4^	10^−5^	10^−6^	10^−7^	10^−8^		
Stem LAMP^a^	F3/B3, FIP/BIP, LF/LB, SF/SB	+	+	+	+	+	*−*	*28*	This study
Stem LAMP^b^	FIP/BIP, LF/LB, SF/SB	*+*	*+*	*+*	+	+	*−*	*28*	This study
Standard LAMP^c^	F3/B3, FIP/BIP, LF/LB	*+*	*+*	*+*	*+*	*−*	*−*	*33*	This study
Standard LAMP^d^	F3/B3, FIP/BIP	*+*	*+*	*±*	*−*	*−*	*−*	–	This study
Published LAMP^e^	F3/B3, FIP/BIP	*+*	*+*	*+*	*−*	*−*	*−*	*39*	Liang et al. [32]
PCR test	EntaF and EhR	*+*	*+*	*+*	*−*	*−*	*−*	*nd*	Hamzah et al. [20]

The cycle threshold (C_*T*_) in minutes for tenfold dilution of 10^−5^ (3 ng/ml)

Neat = ~300 µg/µl

*nd* not done

^a^LAMP test with outer F3/B3 primers

^b^LAMP test without outer F3/B3 primers (amplicons are less bright)

^c^Standard LAMP test (the most common LAMP format)

^d^Standard LAMP test format without loop primers (initial format, not commonly used)

^e^Published LAMP test without loop primers

± Half of the replicates were positive (2 out of 4)

The lower the value the greater the amount of target DNA formed (in italics)

## Results

### *E. histolytica* LAMP optimum reaction conditions

The Taguchi method determined the optimal concentrations for the four reaction components in stem 18S LAMP test as 35 pmol for FIP/BIP, 18 pmol for loop primers, 23 pmol for stem primers and 2 mM dNTPs. The stem HLY6 LAMP test showed the most efficient reaction at 40 pmol for FIP/BIP, loop primers at 20 pmol, stem primers at 15 pmol and 1.5 mM dNTPs. Concentrations for other reagents were as reported previously [22]. The optimum temperature for stem LAMP test was determined at 62 °C and 50 min being the reaction cut-off point. Stem18S LAMP test indicated superior sensitivity to stem HLY6 LAMP test hence the latter was not progressed in the analysis of clinical samples.

### *E. histolytica* LAMP product

**Fig. 1:**
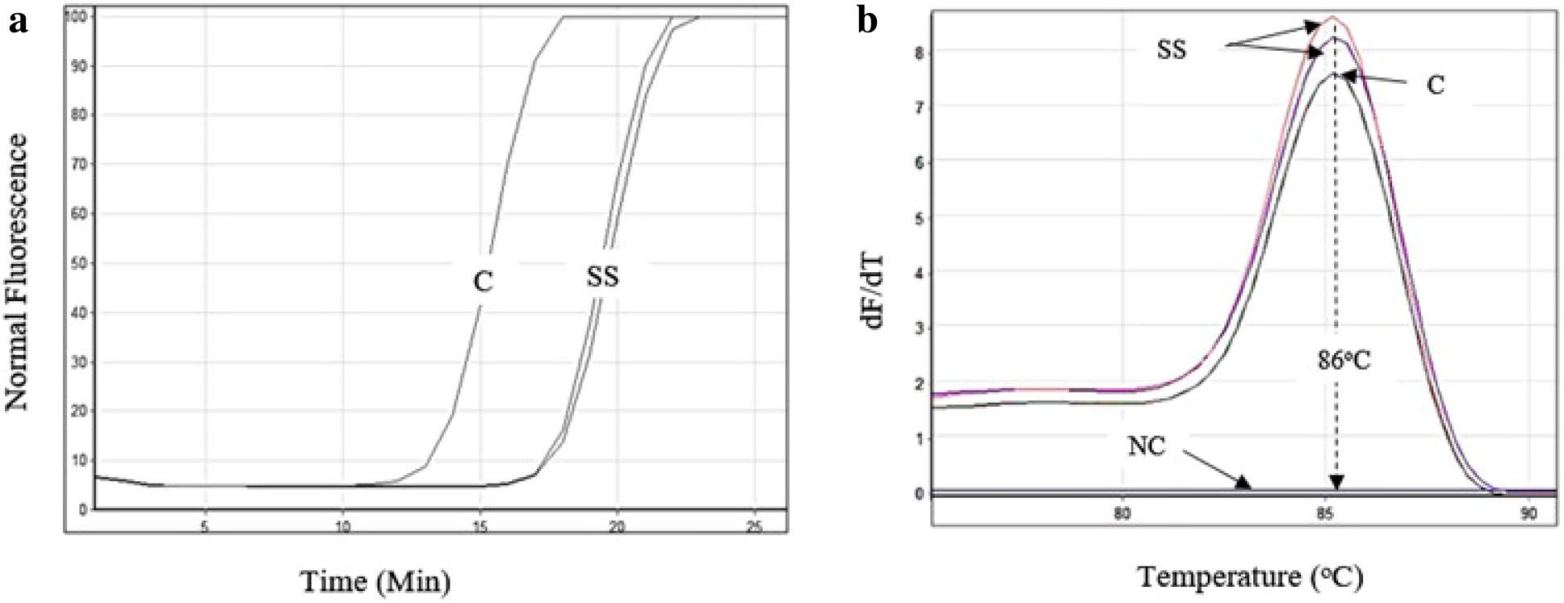
**a** The real-time curves acquired using *E. histolytica* stem 18S LAMP test as monitored using the real time PCR machine. The stem LAMP tests with and without outer primers (F3 and B3) showed similar amplification curves. **b** The *E. histolytica* melt peaks acquired post amplification on the FAM channel. The positive clinical samples showed identical *T*_*m*_ of ~86 °C with the reference DNA indicating identical amplicons. *C* positive control DNA, *SS* two samples using PCR, *NC* negative control, *dF/dT* fluorescence

**Fig. 2:**
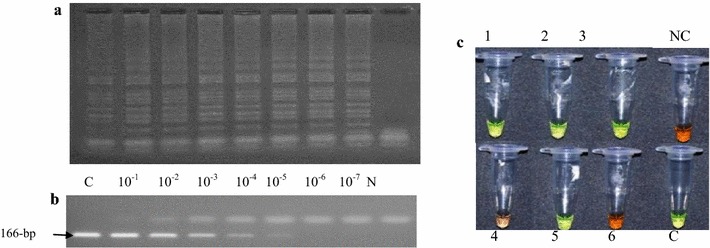
**a** The PCR sensitivity levels and showing the 166 bp amplicon using a tenfold serial dilution of *E. histolytica* DNA. **b** The sensitivity of the stem 18S LAMP test with outer primers. The LAMP amplicons were less bright with the forma without outer primers F3/B3 (image not shown) but the sensitivity levels were identical. **c** The visual appearance of stem 18S LAMP test amplification product after addition of 1/10 dilution of SYBR^®^ Green I dye. The dye fluoresces strongly when bound to the double stranded DNA and the resulting DNA-dye-complex gives a green colour while fluorescence is minimal when the dye is free in the solution and gives orange/brown colour. Samples 1, 2, 3 and 5 are *E. histolytica* positive and samples 4 and 6 are negative samples. *C* control, *N* negative control

The optimized *E. histolytica* stem 18S LAMP tests with and without outer primers indicated similar exponential real time amplification curves (Fig. 1a) with post amplification melting temperature (*T*_m_) of ~86 °C (Fig. 1b). The LAMP products showed the ladder like pattern on the agarose gel indicating the formation of stem-loop with inverted repeats (Fig. 2b). On addition of SYBR^®^ Green I, the positive product turned green and the negative ones remained orange (Fig. 2c). The *Dde*I restriction enzyme digestion of stem 18S LAMP test product indicated the predicted amplicons of 143 and 103 bp.

### Analytical sensitivity of LAMP and PCR tests

The stem 18S LAMP tests (with and without outer primers) indicated identical detection limit of 10^−7^ (30 pg/ml) (Fig. 1a; Table 2) while the standard LAMP test (with loop primers) and published LAMP test (without loop primers) indicated detection levels ranging from 30 to 300 pg/ml (Table 2). The standard LAMP test (without loop primers) showed low sensitivity and was not include in further analysis (Table 2). The stem 18S LAMP test sensitivity was not altered when the stem primers were used either in their forward or reverse orientation and/or when the template was increased from 2 to 4 µl. The PCR test based on the same target showed detection limit of 10^−5^ (3 ng/ml) (Fig. 1). The stem 18S LAMP test sensitivity was reproducible using thermocycler and water bath and no cross reactivity was recorded with non-target DNA. The optimized *E. histolytica* stem 18S LAMP test with and without outer primers F3 and B3 showed reduction in reaction time (cycle threshold = C_*T*_) value of ~11 cycles (Table 2) compared to the standard LAMP test targeting the same gene.

### Results for clinical samples

The stem 18S LAMP tests with and without outer primers detected 36 (28.6%) while the standard and published LAMP tests detected 26 (20.6%) and 21 (16.7%) of *E. histolytica* DNA from samples scored as *Entamoeba* spp. using microscopy respectively (Table 3). We recorded intermittent non-specific products with some replicates for stem LAMP test with outer primers, in which case the replicates were repeated. The conventional PCR classified 18 (14.3%) as *E. histolytica*. Other LAMP tests formats were not used in sample analysis since they indicated inferior analytical sensitivity.

**Table 3:** Comparative evaluation of stem-18S LAMP, standard and published LAMP test and PCR in detection of *E. histolytica* DNA in clinical samples (n = 126)

Type of test	Accelerating primers	No. positive	Reference
Stem 18S LAMP	Loop and stem	36 (28.6%)	This study
Standard LAMP	Loop	26 (20.6%)	This study
Published LAMP	none	20 (15.9%)	Liang et al. [32]
PCR^a^	n/a	18 (14.3%)	Hamzah et al. [20]

^a^PCR positive samples were positive using all LAMP formats

## Discussion

In the present study we have designed a rapid and visual LAMP assay for detection of *E. histolytica*. The stem18S LAMP test is a modification of the standard LAMP test through inclusion of stem primers and indicate superior analytical sensitivity and shorter reaction time to results and translate to a higher detection of pathogen DNA in clinical samples compared to the standard LAMP format (Tables 2, 3). The recorded superior sensitivity can be attributed to the multiplexing of two reaction accelerating primers (loop and stem primers) in a single reaction as compared to the standard LAMP format with and/or without loop primers. The loop primers accelerate the reaction by priming the sequence loops between FIP/BIP primers [37] while the stem primers accelerate reaction by targeting the stem section of the sequence [35]. It is therefore the use of two reaction accelerating primers that exponentially increase the amount of LAMP product, hence reduction in reaction time and increase in sensitivity. Surprisingly the omission of outer primers did not affect the stem 18S LAMP test sensitivity, although the ladder like bands on agarose gel were less bright compared with the format with the outer primers. This may indicate formation of less product in the latter format but did not translate to less sensitivity in terms of pathogen DNA detection. Indeed, the products of the two LAMP formats were confirmed to be identical through acquisition of post amplification melt curves (Fig. 1) and through digestion of the product with restriction enzyme. The primary role of the outer primers is to displace the newly synthesized strands into a single strand making it available for extension by either inner primer [22] and do not form part of the final LAMP product. It appears that the remaining primers may have some strand displacement activity, although not as efficient as the outer primers. The possibility of omitting the outer primers gives more flexibility for positioning of the remaining primers [35].

It is not clear as to why the LAMP test based on the HLY6 gene showed low sensitivity (10^−2^) and low detection of PCR positive samples despite the reported higher number of copies (~400 copies) [33]. One possibility is that the reference DNA and the Kenyan samples may have mutation on the HLY6 gene or on the sequence section targeted by the published primers hence poor priming. Sequencing of the HLY6 gene from Kenyan isolates may answer this question in future. The lower sensitivity of the published LAMP format [32] compared to stem LAMP format is attributable to absence of loop primers. Indeed, our identical LAMP format based on the same gene showed similar lower detection levels with the published format (Table 2). On addition of loop primers, this LAMP format analytical sensitivity improves by tenfold and translate to detection of more positive clinical samples (Table 3). The use of loop primers to accelerate LAMP tests is recommended [37] and has been demonstrated to significantly improve LAMP tests sensitivity and detection of pathogen DNA in clinical samples [42, 43]. The sensitivity of *E. histolytica* LAMP test is further improved in this study through multiplexing loop primers with stem primers. This sequential addition of primers resulting in improvement of LAMP test sensitivity is unequivocal demonstration that the reaction accelerating primers are critical to any successful LAMP test. The resulting product was easily detected using SYBR^®^ Green I dye allowing visual inspection of results. The SYBR^®^ Green I is cheap but the need to open the tube to add the dye risk contamination with amplicon. Further the dye is non-specific and binds to any double stranded DNA including primer-dimers. To increase the confidence of using non-specific dyes, rigorous test optimization is necessary to reduce formation of spurious products. In addition, the use of more negative controls is recommended to the increase the confidence limit.

The stem LAMP test classified 36 (28.6%) of 126 DNA samples as *E. histolytica*. More encouraging results were that all PCR positive samples were also positive with stem 18S LAMP test, indicating that both tests were detecting the same thing. In this study, the detection rate of *E. histolytica* was at 14.4% using PCR and is equivalent to that reported earlier of 13.3% [20]. All LAMP formats showed detection range of 15.9–28.6% which indicates LAMP method is superior to classical PCR and is a good improvement towards diagnosis of amoebiasis. Similar superior sensitivity of stem LAMP format to PCR has been recorded in diagnosis of sleeping sickness [36].

This is the first study in Kenya to report the detection of *E. histolytica* using LAMP method. It is possible that the prevalence of *E. histolytica* is even higher since a large portion of samples remained un-identified and/or that the microscopically observed cysts belong to the morphologically similar but non-pathogenic *E. dispar* and *E*. *moshkovskii.* No tests were done to check the presence of *E. dispar* and *E. moshkovskii*. The world prevalence of *E. dispar* is reported to be nine times that of *E. histolytica* [2]. If that phenomenon holds in the prevalence of this species in Kenya, then a large portion of the remaining 90 (71.4%) DNA could be *E. dispar*. Having methods that can accurately differentiate *Entamoeba* spp. will help estimate their prevalence in Kenya and avoid unnecessary chemotherapy in patients with non-pathogenic species. It should be noted that in amoebiasis, the reason to treat is based on demonstration of trophozoites and/or cysts in the stool, as such LAMP test may not be relied upon to make a treatment decision. Since LAMP test is faster to perform, the technique could form part of diagnostic algorithms for amoebiasis where LAMP test is used to select cases for further confirmation with PCR.

## Conclusions

In this study:

i. A new stem 18S LAMP test which is a modification of the standard LAMP test through inclusion of stem primers was developed.
ii. The stem 18S LAMP test recorded superior sensitivity and shorter reaction time to results.
iii. The detection rate of *E. histolytica* using the new test was higher than prevalence recorded earlier.

It is therefore recommended that this new stem 18S LAMP test be part of diagnostic algorithms for amoebiasis.

## Abbreviations

AIDS: acquired immune deficiency syndrome
ASL: stool lysis buffer
bp: base pair
C_*T*_: cycle threshold
DNA: deoxyribonucleic acid
dNTPs: deoxyribonucleotide triphosphates
EDTA: ethylenediaminetetraacetic acid
ELISA: enzyme-linked immunosorbent assay
g: gram
HIV: human immunodeficiency virus
HLY6: hemolysin 6 gene
KEMRI: Kenya Medical Research Institute
LAMP: loop-mediated isothermal amplification
ml: milliliter
mM: millimolar
NTD: neglected tropical disease
PCR: polymerase chain reaction
Pmol: picomole
qPCR: quantitative PCR
RNA: ribonucleic acid
rRNA: ribosomal RNA
T_m_: melting temperature
U: unit
UV: ultra violet
V: volts
WHO: World Health Organization
